# Oral Pyogenic Granuloma: A Case Report

**DOI:** 10.7759/cureus.116214

**Published:** 2026-09-14

**Authors:** Durgadevi Pancharethinam, Karthik Rajaram Mohan, Saramma Mathew Fenn, Mohithan Subramaniam, Bharath C

**Affiliations:** 1 Oral Medicine and Radiology, Sree Balaji Dental College and Hospital, Bharath Institute of Higher Education and Research (BIHER), Salem, IND; 2 Oral Medicine and Radiology, Vinayaka Mission's Sankarachariyar Dental College, Vinayaka Mission's Research Foundation (Deemed to be University), Salem, IND; 3 Preventive and Community Dentistry/ Public health Dentistry, Vinayaka Mission's Sankarachariyar Dental College, Vinayaka Mission's Research Foundation (Deemed to be University), Salem, IND

**Keywords:** calculus, diascopy, gingiva, pyogenic granuloma, surgical excision

## Abstract

Pyogenic granuloma is a type of lobular capillary hemangioma that bleeds profusely even on slight provocation. In this article, we will discuss the etiopathogenesis, clinical features, differential diagnosis, and treatment of oral pyogenic granuloma.

## Introduction

Pyogenic granuloma is a non-neoplastic tumor-like growth, which was first described by Hullihen in 1884, Poncet and Dor in 1897, named by Crocker in 1903, and introduced by Hartzell in 1904. Clinically, exophytic growth manifests as reddish papules on a pedunculated base. The various etiologies proposed for its formation include chronic low-grade irritation from calculus resulting from poor oral hygiene, traumatic injuries, drugs such as cyclosporine and indomethacin, botryomycosis hominis, *Porphyromonas gingivalis *infections, and pregnancy, which contribute to its development. More than 75% of oral pyogenic granulomas involve the labial aspect of marginal gingiva in the maxillary anterior region, and few involve the gingiva's facial and lingual aspects in between the teeth. The term "pyogenic granuloma" is a misnomer as the term "pyogenic" does not represent "pus" or " granuloma" nor does it represent a granulomatous inflammation [1-4].

## Case presentation

A 32-year-old female patient presented with a chief complaint of bleeding from her gums while brushing her teeth in the upper front tooth region for the past two months. A general examination revealed that her vitals were stable and that there was no pyrexia. Extraoral examination revealed no cervical lymphadenopathy. On intraoral examination, there was an exophytic growth, measuring approximately 1 x 1.5 cm in diameter, involving the labial aspect of free or marginal gingiva between the right maxillary central and lateral incisor teeth. The growth surface appeared shiny, reddish in the center and pale pink near the periphery (Figure 1). Increased interdental spacing was present between the right maxillary central and lateral incisors.

**Figure 1:**
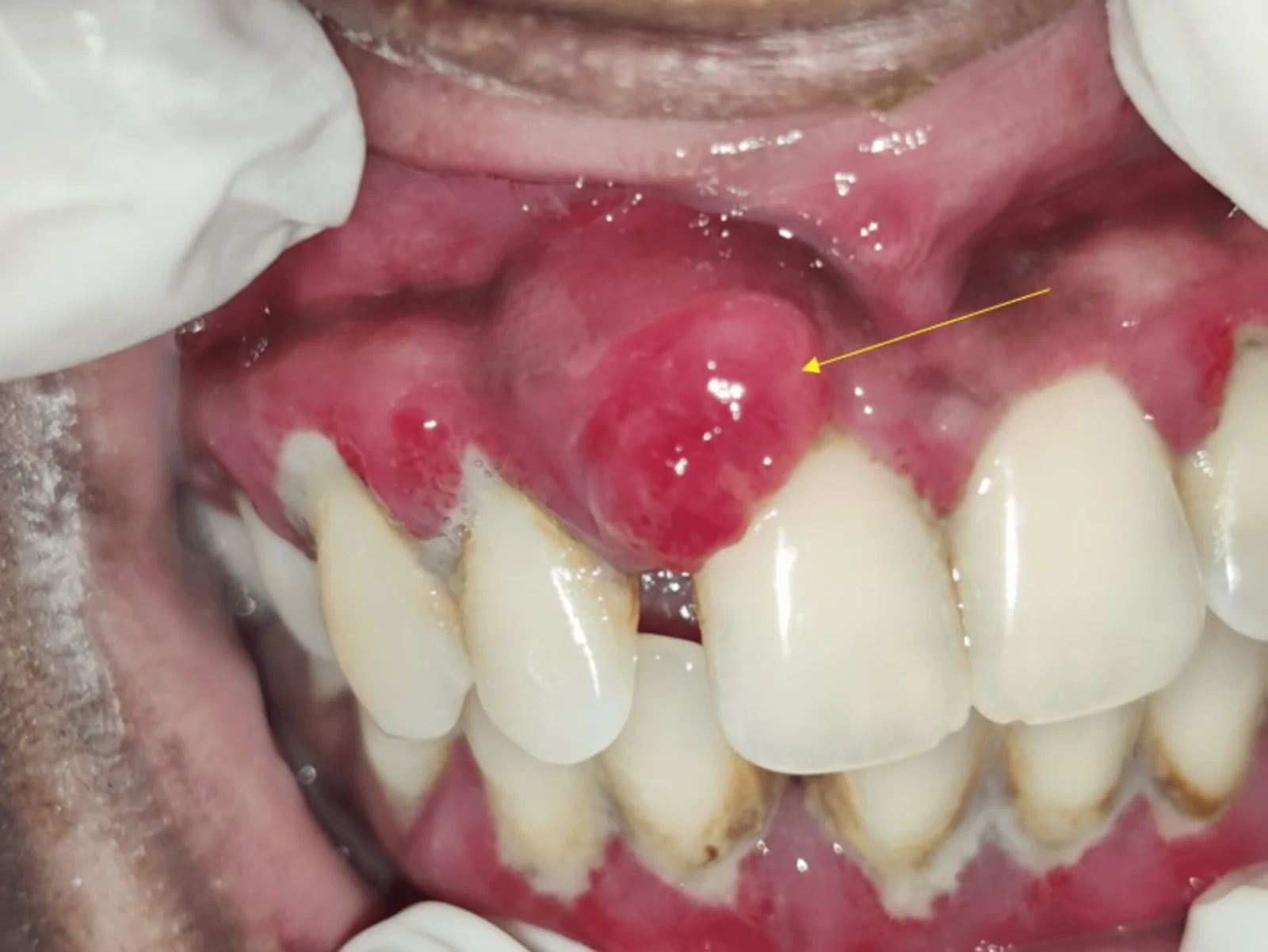
Intraoral examination revealed a growth involving the marginal and interdental papilla between teeth 11 and 12

An angular bone loss was discovered on the intraoral periapical radiograph between the right maxillary central and lateral incisor tooth region. The reason for the angular bone loss was poor oral hygiene, which resulted in calculus deposits near the cementoenamel junction of the teeth, which caused and triggered the periodontal disease resulting in bone loss (Figure 2).

**Figure 2:**
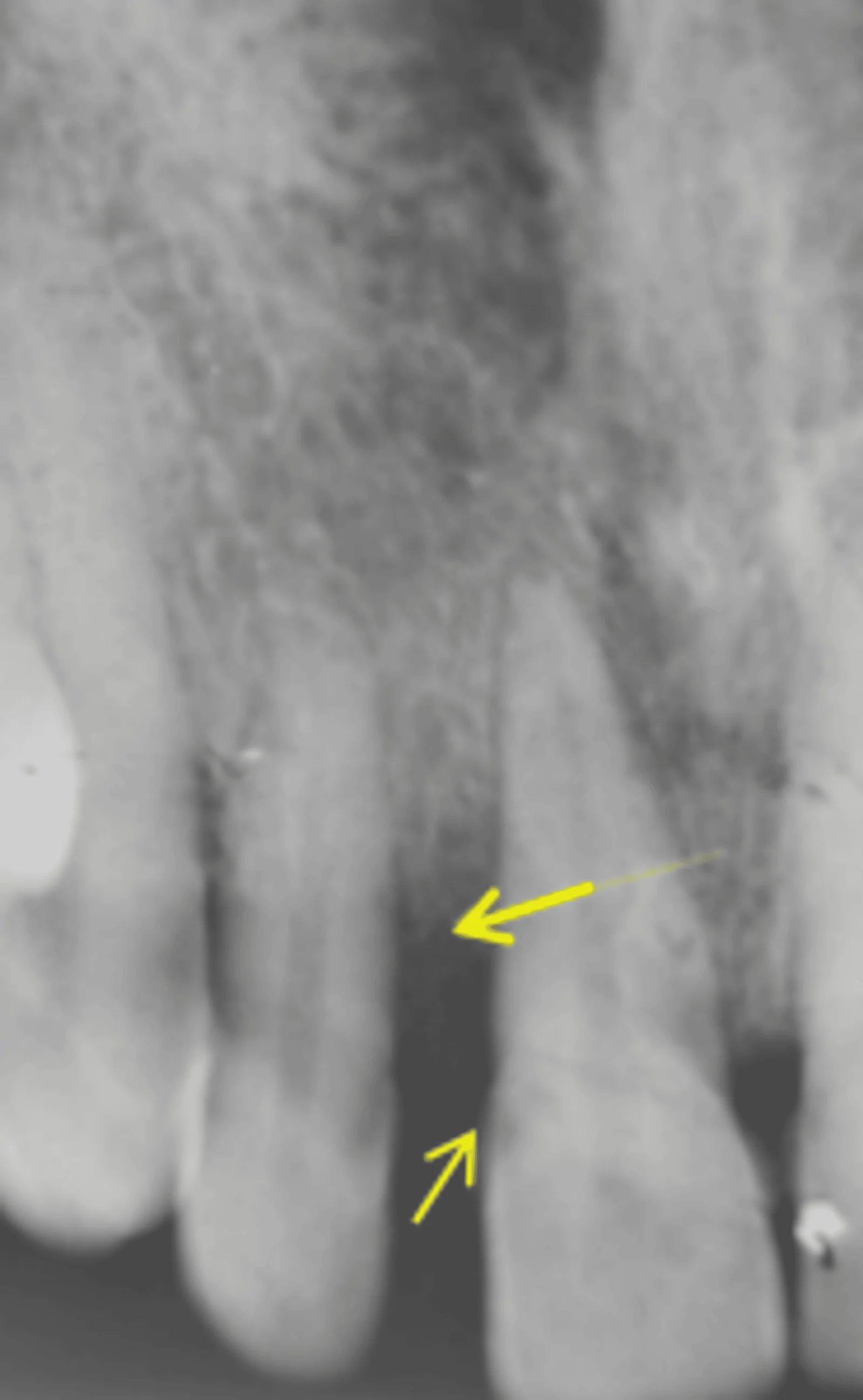
Intraoral periapical radiograph revealed an angular bone loss between teeth 11 and 12 and radiopaque flecks, suggestive of calculus near the mesioproximal crown portion of teeth 11 near the cementoenamel junction (yellow arrow)

Subgingival calculus harbours pathogenic microorganisms that colonize and grow continuously at the junctional epithelium attachment of the gingival sulcus and the underlying alveolar bone. The living bacteria attached to the calculus release endotoxins (lipopolysaccharides) into the adjacent tissue. The host immune system reacts by releasing local inflammatory mediators, specifically pro-inflammatory cytokines interleukin-1 (IL-1), interleukin-6 (IL-6), tumor necrosis factor-alpha, prostaglandins (PGE2), and receptor activator of nuclear factor-kB ligand. These inflammatory chemokines shift the balance of normal bone remodeling. They stimulate bone progenitor cells to mature into osteoclasts, resulting in the destruction of bone and subsequent bone loss. The bacterial biofilm on a calculus deposit has an effective tissue destruction of 1.5-2.5 mm. Bone resorption occurs as a protective attempt by the host to move away from the site of localized bacterial infection. The growth was pedunculated, firm in consistency, and non-tender on palpation, but bled even on slight provocation. A provisional diagnosis of fibroma was made based on the history and clinical findings. The differential diagnoses were pyogenic granuloma, inflammatory gingival enlargement, drug-induced gingival enlargement, Kaposi's sarcoma, and capillary hemangioma. The diascopy test was performed as the lesion appeared reddish. The growth showed a positive response to the diascopy test (Figure 3).

**Figure 3:**
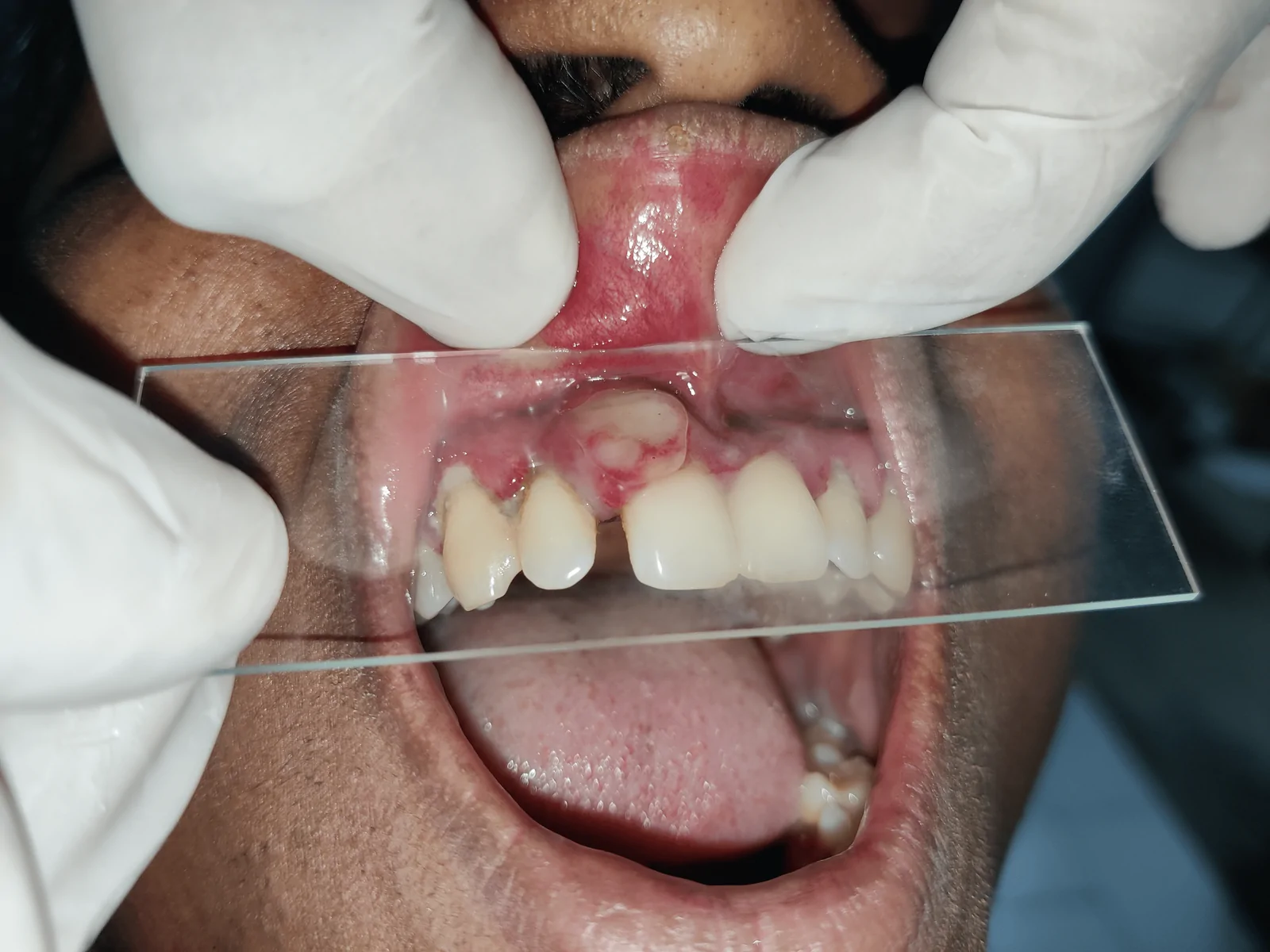
Diascopy test

A peripheral blood smear revealed neutrophils of 6,860 cells, lymphocytes of 3,816 cells per microliter of blood, a bleeding time of 8 minutes, and a clotting time of 12 seconds, which were within biological ranges. The lesion was surgically excised under local anesthesia. The excised specimen is shown in Figure 4.

**Figure 4:**
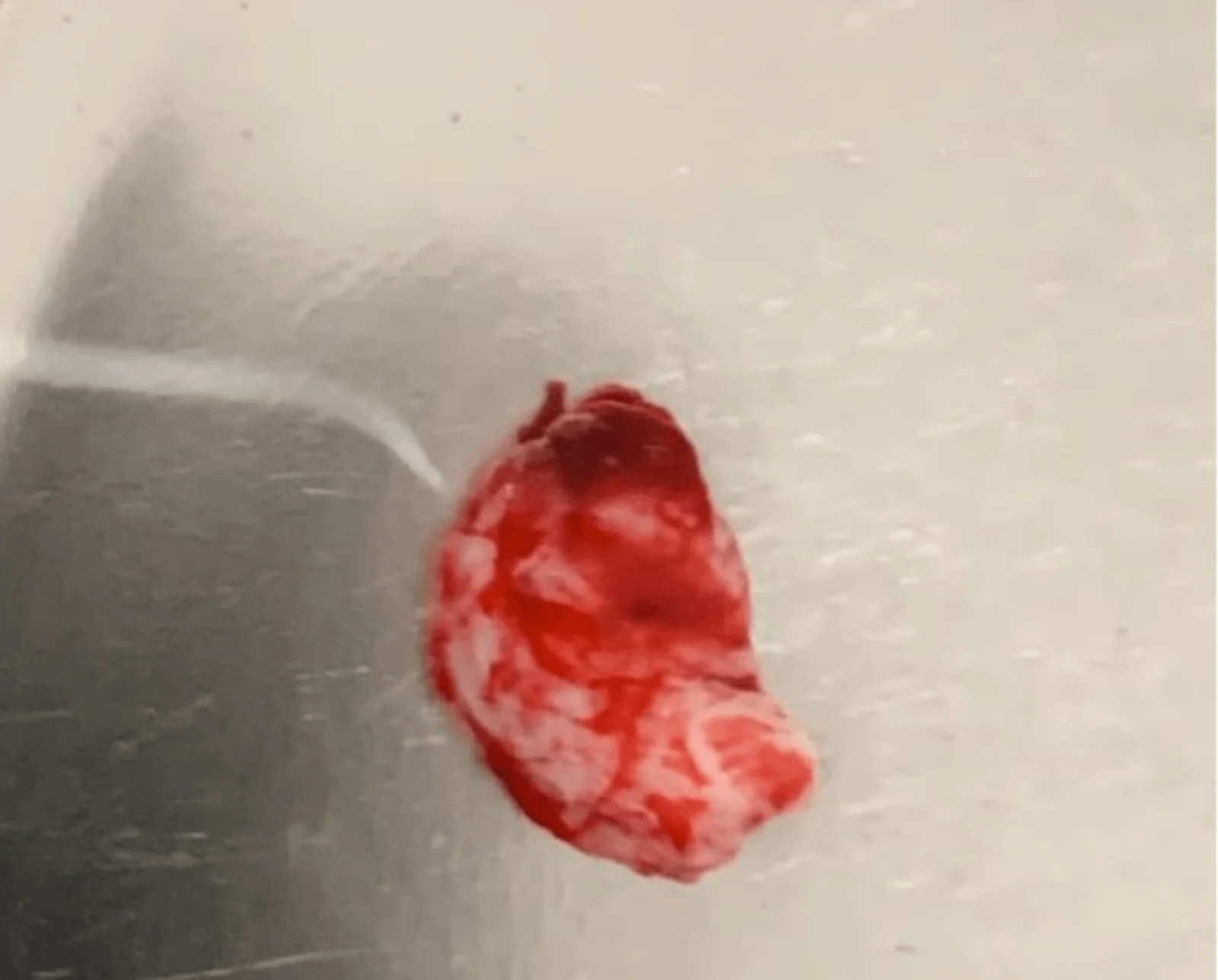
Surgically excised biopsy specimen

No recurrence was reported after surgical excision. The histopathological photomicrograph under high power (x200 magnification) revealed numerous capillary blood vessels (Figure 5).

**Figure 5:**
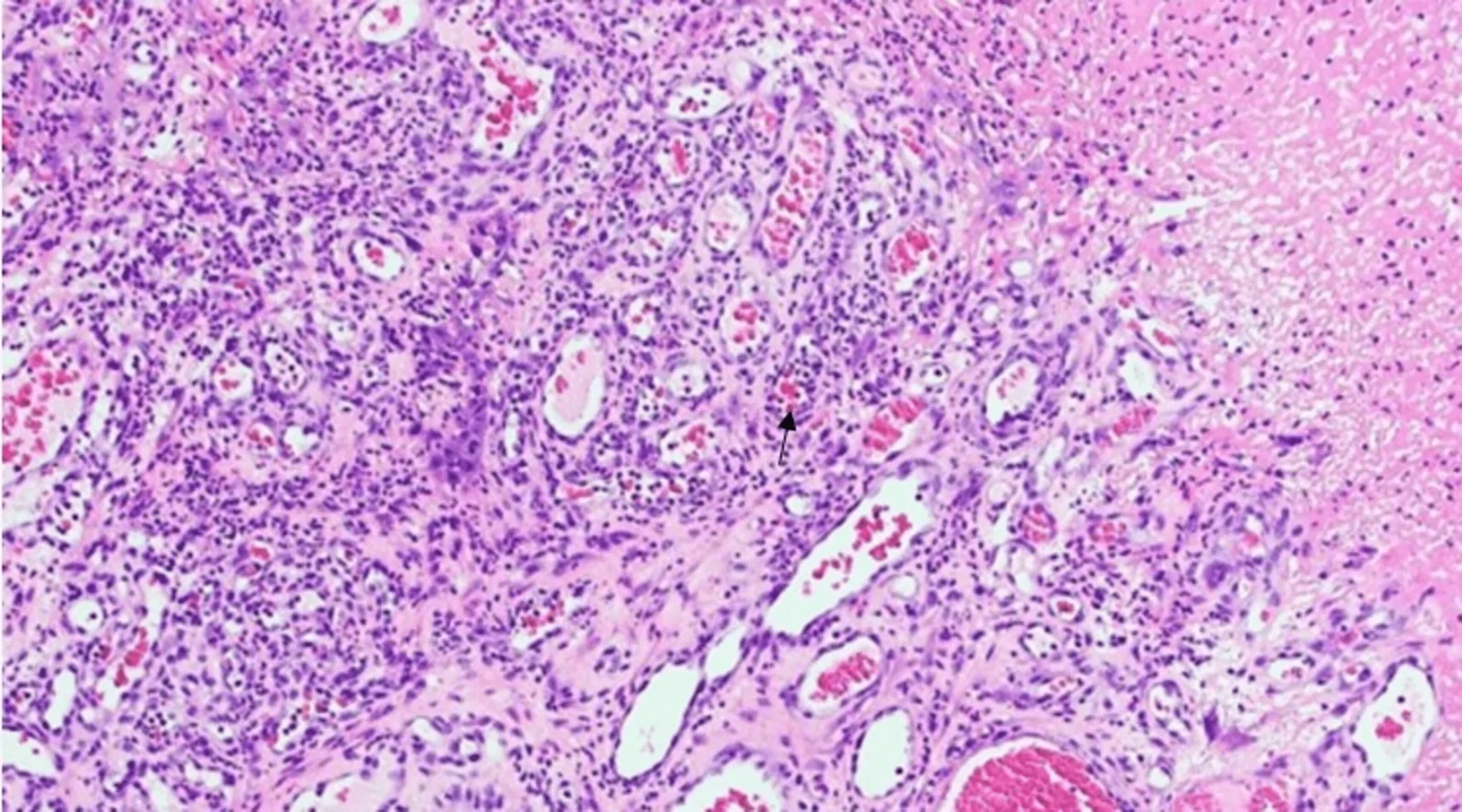
Histopathological photomicrograph revealed numerous capillaries (black arrow)

## Discussion

Definition

Pyogenic granuloma is a non-neoplastic, acquired, benign vascular proliferation of capillaries lined with a single layer of endothelium in tissues on mucosal or skin surfaces [1].

Synonyms

Pyogenic granuloma is also known as Crocker and Hartzell's disease, lobular capillary hemangioma, vascular epulis, hemangiomatous granuloma, granuloma pediculatum benignum, granuloma pyogenicum, pregnancy epulis, granuloma gravidarum [5-7].

Etiological factors

The etiologies of oral pyogenic granuloma are diverse and controversial. Proposed etiological factors include chronic irritation by calculus from poor oral hygiene, traumatic injuries such as basketball injuries, trauma from mucogingival surgery during alveolar socket preservation, hormonal factors such as pregnancy, immunosuppressants such as cyclosporine, drugs such as capecitabine and indomethacin, antineoplastic drugs such as capecitabine and 5-fluorouracil, BRAF inhibitors such as vemurafenib and encorafenib, monoclonal antibodies against epidermal growth factor receptor (EGFR) inhibitors such as cetuximab and panitumumab, and orally active (EGFR) inhibitors such as lapatinib, osimertinib, erlotinib, afatinib, gefitinib, and tyrosine kinase.

Epidemiology

Pyogenic granuloma occurs more commonly among females than males due to the angiogenic proliferation of capillaries induced by increased vascular endothelial growth factor (VEGF) mRNA expression by estrogen and progesterone hormones during parturition or pregnancy. The male-to-female ratio is 1:1.5 [8].

Clinical features

Pyogenic granulomas are common in children and young adults, but they can appear at any age. Because of the vascular effects of the estrogen hormone, there is a greater preference for women. The first trimester of pregnancy is when pyogenic granulomas typically develop, and their frequency rises through the seventh month. The steady rise in the development of these lesions during pregnancy may be linked to rising amounts of progesterone and estrogen. Some of these pyogenic granulomas either go away on their own after delivery and the return of normal hormone levels or develop fibrous tissue and resemble fibromas. Usually, they develop as a growth on the gingiva and appear as pink or red soft masses that are usually pedunculated. However, some are sessile, which bleed on slight provocation due to their excess vascularity. Young pyogenic granulomas appear more reddish on the surface, while older pyogenic granulomas are more collagenous and appear pinker in color. They range in size from small lesions that are a few millimeters across to larger lesions that may be several centimeters across. Usually, the mass is painless. Pyogenic granulomas grow quickly, which may frighten the patient who worries that the lesion might be cancerous. About 75% of pyogenic granulomas occur near the gingiva in the oral cavity. Numerous patients complained of no or mild discomfort and inflammation brought on by calculus from inadequate oral hygiene. The next common sites include the lips, tongue, hard palate, and buccal mucosa. Extraoral pyogenic granuloma can occur in the esophagus, epiglottis, retina of the eye, and gastrointestinal tract, involving the small intestine, duodenum, and proximal part of the nail unit. They range from a few millimeters to greater than 7 cm in diameter [9-11].

Clinical variants

Pyogenic granuloma can present in several distinct clinical variants. Digital or periungual pyogenic granulomas may occur as solitary or multiple lesions and are associated with chronic skin conditions such as atopic dermatitis and psoriasis [1]. Disseminated pyogenic granulomas usually develop after burns, following isotretinoin therapy for nodulocystic acne, or after treatment with systemic granulocyte colony-stimulating factor (G-CSF) [1]. Satellite pyogenic granulomas typically develop following surgical excision of a primary lesion [1]. Ossifying pyogenic granuloma is an uncommon variant that occurs on or adjacent to the proximal nail bed; these lesions undergo osseous metaplasia and contain osteoclastic giant cells [12].

Differential diagnosis

Kaposi's sarcoma clinically mimics pyogenic granulomas. Still, such patients have seropositivity for human herpes virus (HHV-8) DNA sequences targeted by polymerase chain reactions (PCRs), which do not occur in pyogenic granuloma [13,14]. Fibroma on the gingiva probably represents pyogenic granulomas that have undergone fibrous maturation [15]. Hyperplastic granulation tissue growths that occasionally appear in healed extraction sockets are known as epulis granulomatosa. These lesions typically show a granulation tissue response to the bone sequestra in the socket and frequently mimic pyogenic granulomas [15]. Inflammatory gingival enlargement occurs involving the marginal and attached gingiva. Chronic inflammatory enlargement is characterized by the presence of calculus resulting from poor oral hygiene. Pyogenic granulomas in the younger age group appear as more reddish enlargements due to their high vascularity, while pyogenic granulomas in the older age group characteristically appear as more pinkish growths due to the highly fibrotic nature of such lesions. Chronic inflammatory gingival enlargement does not bleed much like pyogenic granulomas [15]. Drug-induced gingival enlargement is associated with a positive history of medications such as the antiepileptic drug phenytoin or antihypertensive agents such as amlodipine and nifedipine. These drugs cause diffuse overgrowth of the marginal gingiva and interdental papillae, covering the bulbous crowns and producing pseudopockets, reflecting their systemic effect on the gingival tissues. This diffuse, generalized involvement contrasts with pyogenic granuloma, in which the lesion is typically localized to a single site, usually on the gingiva [15].

Histopathological features

Pyogenic granulomas exhibit highly vascular growth that resembles granulation tissue when examined under a microscope. Red blood cells are arranged into several tiny and bigger endothelium-lined channels. Sometimes these veins are clustered in lobular aggregates, which help histopathologists make a diagnosis. A mixed inflammatory cell infiltrate of neutrophils, plasma cells, and lymphocytes is evident. Neutrophils are most common near the skin's surface, while cells that cause chronic inflammation are found deeper [15].

Immunological factors

Immunohistochemical studies of pyogenic granuloma have demonstrated increased expression of several vascular morphogenesis factors, including endothelial growth factor, angiopoietin 1 and 2, ephrin B2 and B4, and basic fibroblast growth factor (FGF), along with positivity for anti-CD34 antibodies and factor VIII antigen. Increased expression of nitric oxide synthase, Bcl-2 and Bax proteins, and phosphorylated mitogen-activated protein kinase has also been reported [16].

Treatment and prognosis

Surgical procedures are used to treat patients with pyogenic granulomas because they are benign and do not have the potential to become cancerous. The neighbouring teeth should be scaled to remove any potential sources of persistent irritation after the excision, which should be performed down to the periosteum. Sometimes, the lesion returns, necessitating further excision. Multiple recurrences are only sometimes noted. Surgical excision treatment should be deferred for lesions that develop during pregnancy unless significant aesthetic and functional problems exist. Since the recurrence rate of such pyogenic granulomas is higher during pregnancy, some pyogenic granulomas resolve spontaneously after parturition. The other treatment modalities for pyogenic granuloma include water-cooled diode lasers, Er: YAG lasers, long-pulsed 1,064 nm Nd: YAG lasers, cryotherapy, sclerotherapy using intralesional ethanolamine oleate, polidocanol, sodium tetradecyl sulphate, bleomycin, by topical application with imiquimod, timolol, propranolol, alitretinoin, and electrocautery [17-19].

Complications

The complications of untreated pyogenic granuloma are bleeding from trauma, secondary infections from ulcers, psychological distress from disfigurement if pyogenic granuloma occurs in the face, and cancerophobia because of spontaneous bleeding from single endothelium lined capillaries, which are highly prone to fragility [20].

## Conclusions

Pyogenic granuloma is a perpetual lesion that can confuse the clinician due to increased bleeding on even slight provocation. A careful clinical examination and diascopy - a chair-side diagnostic test - help in the early diagnosis of such lesions. Constant irritation due to calculus resulting from poor oral hygiene was the chief cause of the pyogenic granuloma in our case. Proper oral brushing techniques and hygiene instructions are necessary to prevent pyogenic granuloma. Counselling and reassurance must be provided for such affected patients with pyogenic granuloma to alleviate the fear of cancerophobia. Programs that teach people how to keep their oral cavity clean by using the correct brushing techniques and oral hygiene practices can help reduce the number of cases of this common threat, which is often seen in clinics and among patients.
